# Strategies for poverty alleviation supply chain with government subsidies and misreporting behavior in China

**DOI:** 10.1371/journal.pone.0253761

**Published:** 2021-07-15

**Authors:** Fangyu Ye, Qilong Deng

**Affiliations:** 1 School of Business Administration, Hunan University, Changsha, Hunan Province, PR China; 2 School of Business, Central South University of China, Changsha, Hunan Province, PR China; Szechenyi Istvan University: Szechenyi Istvan Egyetem, HUNGARY

## Abstract

In the poverty alleviation supply chain, subsidies for enterprises or farmers are widely implemented as part of government policy. However, subsidy fraud often occurs, such as misreporting cost information to secure subsidies. Inspired by this, our study aims to explore the optimal decision-making problem of the three-level (government + enterprises + farmers) poverty alleviation supply chain under asymmetric cost information. Four-stage models are constructed to capture the interactions among these three players. Additionally, numerical examples are used to analyze the implications of key parameters, such as cost coefficient and punitive measures coefficient, on supply chain members’ optimal decision and profit. Our findings demonstrate that both the enterprise and the farmer can obtain maximum profit from the misreporting behavior. Unfortunately, this behavior always damages the profit of other participants and weakens the efficiency of subsidy policy. Moreover, to mitigate the negative implication of misreporting behavior, the government can establish punitive measures to curtail misreporting. Our work provides important policy implications for governments and enterprises. To ensure that more consumers have access to poverty alleviation products, government organizations should prioritize such projects. In addition, the provision of public facilities and technical guidance should be more effective and prompt to share enterprises’ and farmers’ costs. We further recommend that subsidy policies be formulated according to recipients’ performance in poverty alleviation projects, with corresponding supervision and punitive measures. Finally, in cooperating with farmers in poverty alleviation, enterprises should maximize their interests and reduce costs through technological innovation and channel sharing.

## Introduction

In September 2015, world leaders set the United Nations Sustainable Development Goals (17 goals) for a world free of poverty, hunger, disease, and want by 2030. Among these goals, no poverty was listed as the first [1]. Unfortunately, poverty often occurs in remote areas of developing countries due to limited access to market information, transportation, and other resources [2]. According to the World Bank, as of 2018, more than 1.885 billion people, or 26.3% of the world’s population, live below the international poverty line of USD 3.2 per person per day [3]. The Statistical Report of National Economic and Social Development in 2018, published by the National Bureau of Statistics of China, points out that over 16.6 million people in rural areas earned less than 2,300 yuan (USD 375.60) per year [4]. As a major agricultural economy, China’s contribution to agricultural output value to GDP is stable at 9–10%. However, due to the lack of market information, lack of roads, and other factors, many farmers in remote areas are faced with difficulties in selling and cashing out and often fall into poverty. The National Rural Poverty Monitoring survey conducted by the National Bureau of Statistics, forecast a number of 5.51 million people in rural poverty by the end of 2020. How to help rural people get out of poverty and earn more is the most urgent issue in China’s economic development today.

To strengthen the poor’s (such as farmers) subjective initiative in increasing their income, governments, non-governmental organizations, and social enterprises offer tools, financing, and channels to improve agricultural production levels [5, 6]. In general, these financial instruments are implemented directly (such as direct purchasing) or indirectly (such as taxes, subsidies, and regulations) [7]. Funds spent on poverty alleviation in China exceeded 300 billion yuan in 2017; these funds are channeled through subsidies to either enterprises or poor farmers.

The local governments provide subsidies for enterprises depending on their poverty alleviation program budgets [8]. These economic incentives encourage enterprises to carry out poverty alleviation projects in poverty-stricken areas; an example of this is the Suning Group founded in 1996, which invested 500,000 yuan in establishing a poverty alleviation base in Jurong City, Jiangsu Province [9]. After investigating the project and its implementation details, the local government and charities raised 1.15 million yuan to promote the project [10]. Some scholars have also focused on cooperation between enterprises and poor areas, such as Sakarya [11], whose paper studied profit generation and distribution of enterprises that cooperate with the poor. Chen et al. investigated an India-based company, ITC Ltd., which operates the E-Choupal program that helps non-contract farmers identify options available to them [12]. Sodhi and Tang studied the poverty score and how to design supply chain infrastructure models as distributors and suppliers to achieve poverty reduction and income generation goals [13]. Although the above researches have studied issues related to enterprise poverty alleviation, they do not reflect the characteristics of enterprise poverty alleviation behavior in the supply chain model, and thus lack pertinence.

Governments can directly distribute subsidies to poor farmers. For instance, the China Resources Group carried out a poverty alleviation project by investing 385 million yuan and building 10 beef breeding bases in Ningxia Province, China. Two thousand yuan per kgs of beef is provided for poor farmers after the program [14]. Although these poverty alleviation projects achieve fruitful results, some enterprises and farmers use policy loopholes to defraud poverty alleviation subsidies by misreporting cost information [15]. These behaviors seriously influence the promotion of poverty alleviation projects. One example is that, in 2013, farmers in Hunan province misreported the number of cattle and defrauded the poverty alleviation funds by more than 80,000 yuan. These behaviors seriously damage the interests of people genuinely trapped in poverty and need help. An excellent initial idea from the government may not achieve a successful result in the end. Therefore, inspired by these examples, our study explores the optimal decision-making problem of the three-level poverty alleviation supply chain under asymmetric cost information, and intends to solve two main problems. (1) In the process of poverty alleviation programs, how does misreporting behavior affect the design of subsidy mechanisms and supply chain members’ optimal decisions? (2) How should the government design a supervisory and punitive policy to encourage both farmers and enterprises to report real cost information and improve poverty alleviation efficiency? The main contribution of this paper is in providing a management perspective for poverty alleviation subsidy policy design based on socially responsible operations, especially on how to develop an effective subsidy and punitive measures policy under enterprises’ and farmers’ misreporting behavior.

## Methodology

### Model settings and notations

We consider an agricultural supply chain comprising three players: government, enterprises, and farmers. In this supply chain, enterprises purchase crops produced by the farmers and sell these crops to the market. During this process, the government supports either farmers or enterprises in poverty alleviation projects via subsidy policy and other programs [16, 17]. Unfortunately, both farmers and enterprises have incentives to misreport cost information to fraudulently secure poverty alleviation subsidies.

**Fig 1:**
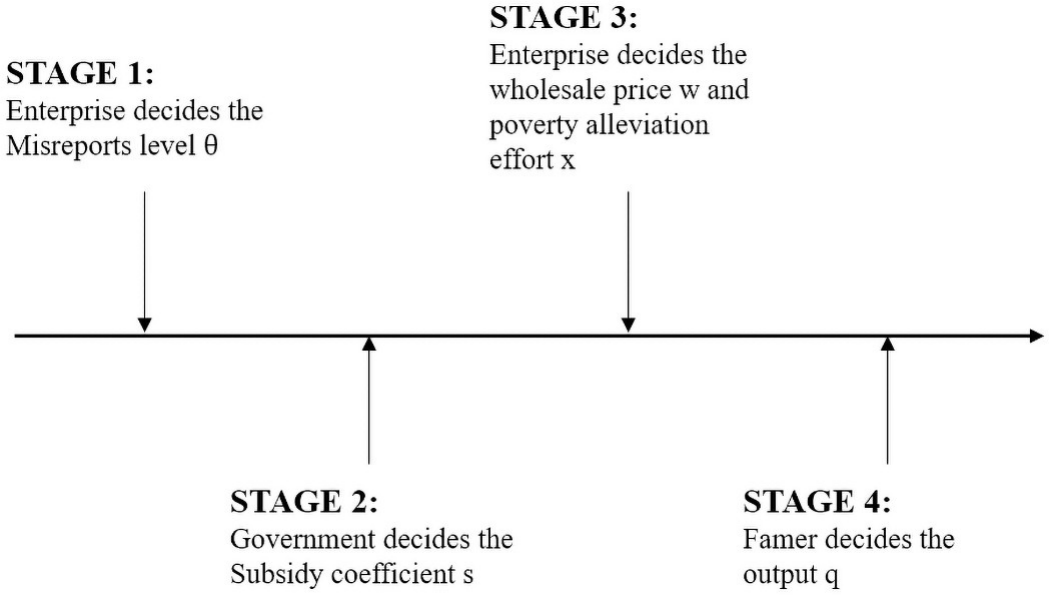
Decision order of supply chain member.

Our study builds a four-stage model to capture these three players’ interactions, as shown in Fig 1. First, the government investigates enterprise poverty alleviation programs in poor areas. At this time, the enterprise or firm decides whether to misreport the corresponding cost information, and the corresponding misreport level is $\theta_{ES}^{E}$. Second, the government determines the subsidy coefficient of poverty alleviation $S_{ES}^{E}$. Third, the enterprise determines the wholesale price of crops *w* and the poverty alleviation effort level *x*. Finally, the farmer determines the total output of crop *q*.

To develop our model, the following assumptions are used in the paper.

#### Assumption 1

Following the notations of Agbo et al. [18], we use *C*(*q*) to represent the production cost of farmers, which is a function of agricultural production (*q*), and satisfies that *C*(0) = 0, *C*(*q*)′ ≥ 0, *C*(*q*)″ ≥ 0. For simplicity, and based on existing research [19, 20], we assume that *C*(*q*) = *ςq*^2^ where *ς* is a positive constant.

#### Assumption 2

When enterprises are involved in poverty alleviation, they need to pay corresponding costs. Based on previous research [21], it is assumed that the cost for poverty alleviation *C*(*x*) is positively related to the efforts spent on poverty alleviation *x* and it satisfies that *C*(0) = 0, *C*(*x*)′ ≥ 0, *C*(*x*)″ ≥ 0, and *C*(*x*) = *Bx*^2^/2 where *B* is the coefficient of investment cost from the enterprise. *C*(*x*) is the total cost of a series of services, such as technology, logistics, and information, when cooperating with farmers in poverty alleviation.

#### Assumption 3

Following Agbo et al. [18], our inverse demand function includes two parts. One is associated with crop yield (denoted as *q*) and is assumed to be *P*(*q*) = *A* − *βq*[22, 23]. Motivated by Wang et al. [24] and Jiang et al. [25], consumers have a purchasing preference for the products of enterprises that undertake social responsibility. In the above equation, *A* is consumers’ WTP for products when the crop supply is zero and *β* represents the crop yield’s coefficient. The other part is relevant to the efforts spent on poverty alleviation by the enterprise. This is because consumers prefer products sold by enterprises with better CSR performance [26, 27]. We assume that *bx* characterizes the impact of the efforts spent on poverty alleviation on the consumer market and stands at *b* ≥ 0 for the marginal impact of the efforts spent on poverty alleviation on additional willingness to pay. Thus, combining the two parts above, the price of the crops is *P*(*q*, *x*) = *A* − *βq* + *bx*.

#### Assumption 4

The government’s goal is to develop an optimal subsidy policy. Borrowing some concepts from the model of government + enterprise + farmers [28, 29], we assume that the total social welfare is linear additive, which can be expressed as: where *SW* = *CS* + *PS* − *TS*, $CS=\int_{A+bx-\beta q}^{A+bx}\frac{A+bx-P}{\beta }dP=\frac{\beta q^{2}}{2}$ represents consumer surplus, *PS* = *π*^*F*^ + *π*^*E*^ captures producer surplus. Besides, *TS* is the total subsidy of the government.

The symbols used in the following content are listed in Table 1. The decision variables used in the following content are shown in Table 2. In addition, superscripts *F*, *E*, *and G* represent farmers, enterprises, and governments, respectively. *O* and *L* represent the supply chain’s report and misreport the real cost information, respectively.

**Table 1:** Symbol description.

Symbol	Description
*ς*	Production cost of the farmer, including inputs, time, energy, and other factors
*B*	Coefficient of the enterprise’s investment cost
*β*	Price sensitivity factor of consumer demand
*b*	Consumer’s price sensitivity factor to poverty alleviation efforts
*A*	Potential market
*γ*	Punitive measures coefficient of government
*k*	Level of government supervision over the enterprise (0 < *k* ≤ 1)
*π*	Real profit of supply chain members
∏	Public profit of supply chain members

**Table 2:** Decision variables description.

Decision variables	Description
*q*	Output of crops.
*w*	Wholesale price of crops.
*x*	Poverty alleviation efforts of the enterprise—the larger the *x*, the stronger the corresponding poverty alleviation work of the enterprise. When *x* = 0, it means that the enterprise does not participate in poverty alleviation work.
*θ*	Level of cost information misreporting
*s*	Government subsidy coefficient.

Four models with and without government subsidies and a punitive measures policy are built to analyze the poverty-alleviation supply chain. For notational convenience, the subscript *NS* refers to the NS model without government subsidies (both for farmer and enterprise) and punitive measures policy, *ES* refers to the ES model with government subsidies (only for enterprise) and without punitive measures, *FS* refers to the FS model with government subsidies (only for farmers) and without punitive measures, *SP* refers to SP model with government subsidies (both for farmer and enterprise) and with punitive measures policy. The description of the subscripts is listed in Table 3.

**Table 3:** Subscripts description.

Subscripts	Subsidy policy	Punitive measures policy	Subsidy subject
*NS*	No	No	No
*ES*	Yes	No	Enterprise
*FS*	Yes	No	Farmer
*SP*	Yes	Yes	Enterprise and Farmer

### NS model

In the scenario without government subsidies and punitive measures policy, a two-stage supply chain model composed of farmers and enterprises is established. The purchase cost of agricultural products per unit is *w*. The reverse induction method was adopted to solve this problem. Farmers determine the optimal output of agricultural products. The decision-making problem of farmers is

$$Max\left(\pi_{NS}^{F}\right)=wq-C\left(q\right)=wq-\varsigma q^{2}$$ (1)

$\pi_{NS}^{F}$ is farmers’ profit, whose first-order conditions with respect to *q* are $\frac{\partial \pi_{NS}^{F}}{\partial q}=w-2q\varsigma =0$. Then, we get $\partial^{2}\pi_{NS}^{F}/\partial q^{2}=-2\varsigma <0$. The farmer’s profit function is a concave function with respect to *q*, and the optimal agricultural output satisfies $q_{NS}^{*}=w/2\varsigma$. When the information on poverty alleviation cost is asymmetric, the public profit and the actual profit of the enterprise are as follows:

$$\prod_{NS}^{E}=\left(A+bx-\beta q-w\right)q-\frac{\theta_{NS}^{E}Bx^{2}}{2}$$ (2)

$$\pi_{NS}^{E}=\left(A\text{+}bx-\beta q-w\right)q-\frac{Bx^{2}}{2}$$ (3)

First, the enterprise decides the price of agricultural products *w* and the effort level *x*. Therefore, we calculate the heather matrix of $\prod_{NS}^{E}$:

$$H\left(w,x\right)=\left[\begin{array}{cc} \frac{\partial^{2}\prod_{NS}^{E}}{\partial w^{2}} & \frac{\partial^{2}\prod_{NS}^{E}}{\partial w\partial x}\\ \frac{\partial^{2}\prod_{NS}^{E}}{\partial x\partial w} & \frac{\partial^{2}\prod_{NS}^{E}}{\partial x^{2}} \end{array}\right]=\left[\begin{array}{cc} -\frac{\beta +2\varsigma }{2\varsigma^{2}} & \frac{b}{2\varsigma }\\ \frac{b}{2\varsigma } & -\theta_{NS}^{E}B \end{array}\right]$$ (4)

If $\theta_{NS}^{E}B>\frac{b^{2}}{2\left(\beta +2\varsigma \right)}$ available for a negative semidefinite matrix, which applies to the situation where the profit is negative. Thus, the best decisions of both parties are as follows:

$$w_{NS}^{*}=\frac{2\theta_{NS}^{E}AB\varsigma }{-b^{2}+2\theta_{NS}^{E}B\beta +4B\varsigma }$$ (5)

$$x_{NS}^{*}=\frac{Ab}{-b^{2}+2\theta_{NS}^{E}B\beta \text{+}4\theta_{NS}^{E}B\varsigma }$$ (6)

$$q_{NS}^{*}=\frac{A\theta_{NS}^{E}B}{-b^{2}+2\theta_{NS}^{E}B\beta +4\theta_{NS}^{E}B\varsigma }$$ (7)

By substituting Eqs (4), (5) and (6) into the profit function of farmers and enterprises, the profits of both parties are as follows:

$$\pi_{NS}^{F^{*}}=\frac{{\left(\theta_{NS}^{E}\right)}^{2}A^{2}B^{2}\varsigma }{{\left(-b^{2}+2\theta_{NS}^{E}B\beta +4\theta_{NS}^{E}B\varsigma \right)}^{2}}$$ (8)

$$\pi_{NS}^{E^{*}}=\frac{A^{2}B}{-2b^{2}+4\theta_{NS}^{E}B\beta +8\theta_{NS}^{E}B\varsigma }$$ (9)

### ES model

Some scholars pay attention to the issues of cooperation between enterprises and poor farmers. Michelson et al. studied the direct farm program in Nicaragua and pointed out that farmers usually collaborate with Wal-Mart to avoid risk [30]. However, Wal-Mart offers a purchase price lower than that of the traditional channel. Schipmann and Qaim analyzed different supply channels for sweet pepper in Thailand and found that cooperation without contracts was more popular among farmers [31]. London et al. showed how to create value when social enterprises or for-profit enterprises collaborate with the poor (e.g., farmers) [32].

In view of this, it is necessary to consider a model with government subsidies for enterprises. In this situation, the decision-making problems of farmers and the government are stated as follows:

$$Max\left(\pi_{ES}^{F}\right)=wq-\varsigma q^{2}$$ (10)

$$Max\left(\pi_{ES}^{G}\right)=\frac{\beta q^{2}}{2}+\pi_{ES}^{F}+\prod_{ES}^{E}-\theta_{ES}^{E}\cdot s_{ES}^{E}\cdot \frac{Bx^{2}}{2}$$ (11)

To make the decision-making behavior consistent with false behavior, an enterprise’s decision-making needs to maximize public profits $\prod_{ES}^{E}$.


$$Max\left(\pi_{ES}^{E}\right)=\left(A+bx-\beta q-w\right)\cdot q-\frac{\theta_{ES}^{E}Bx^{2}}{2}\cdot \left(1-s_{ES}^{E}\right)$$ (12)

Similarly, the real profit of the enterprise is:

$$\pi_{ES}^{E}=\left(A+bx-\beta q-w\right)\cdot q-\frac{Bx^{2}}{2}\cdot \left(1-\theta_{ES}^{E}s_{ES}^{E}\right)$$ (13)

As the leader of the supply chain, the government makes decisions before enterprises and farmers do. The decision-making variable is the poverty alleviation subsidy $s_{ES}^{E}$. Enterprises are vital participants in poverty alleviation projects, as they determine the purchase price of agricultural products *w* and the efforts *x*. Then, the farmer determines the planting quantity *q* of agricultural products based on the enterprise’s decision. The inverse induction method is used to solve the problem, and the following decision results are obtained:

$$s_{ES}^{E^{*}}=1/3$$ (14)

$$w_{ES}^{*}=\frac{4A\theta_{ES}^{E}B\varsigma }{-3b^{2}+4\theta_{ES}^{E}B\left(\beta \text{+}2\varsigma \right)}$$ (15)

$$x_{ES}^{*}=\frac{3Ab}{-3b^{2}+4{\left(\theta_{ES}^{E}\right)}^{2}B\left(\beta \text{+}2\varsigma \right)}$$ (16)

$$q_{ES}^{*}=\frac{2A\theta_{ES}^{E}B}{-3b^{2}+4\theta_{ES}^{E}B\left(\beta \text{+}2\varsigma \right)}$$ (17)

Under the situation of optimal decision-making, the total subsidy input from the government to the enterprise is

$$TS_{ES}^{*}=\frac{s_{ES}^{E^{*}}\theta_{ES}^{E}Bx^{2}}{2}=\frac{3\theta_{ES}^{E}A^{2}b^{2}B}{2{\left(3b^{2}-4\theta_{ES}^{E}B\left(\beta +2\varsigma \right)\right)}^{2}}$$ (18)

To make this decision-making behavior consistent with the misreporting behavior, the enterprise makes decisions to maximize its public profit rather than its own profits. Based on the concept of corporate social responsibility (CSR), enterprises need to achieve economic goals and bear obligations, and improve public interest during their operation regarding social responsibility as an expectation for the economy, law, ethics, and charity of enterprises [33–35]. At first, the enterprise decides the misreport level $\theta_{ES}^{E}$. When $\theta_{ES}^{E}=1$, the enterprise reports the real cost information and its profit is $\pi_{ESO}^{E}$. When $\theta_{ES}^{E}>1$, the enterprise misreports the cost information, and its profit is $\pi_{ESL}^{E}$. We can then obtain proposition 1:

**Proposition 1**: When $-3b^{2}+4\theta_{ES}^{E}B\left(\beta +2\varsigma \right)>0$, then

1. When $\theta_{ES}^{E}\in (1,\bar{\theta_{ES}^{E}})$, then $\pi_{ESO}^{E}<\pi_{ESL}^{E}$. When $\theta_{ES}^{E}\geq \bar{\theta_{ES}^{E}}$, then $\pi_{ESO}^{E}\geq \pi_{ESL}^{E}$.
2. When $\theta_{ES}^{E}\in \left(1,\theta_{ES}^{E^{*}}\right)$, then $\frac{\partial \pi_{ESL}^{E}}{\partial \theta_{ES}^{E}}>0$. When $\theta_{ES}^{E}\geq \theta_{ES}^{E^{*}}$, then $\frac{\partial \pi_{ESL}^{E}}{\partial \theta_{ES}^{E}}\leq 0$.

The coefficient of misreports cost information satisfies $\bar{\theta_{ES}^{E}}=\frac{3}{2}-\frac{3b^{2}}{8B\beta +16B\varsigma }$, the optimal misstatement coefficient is $\theta_{ES}^{E*}=\frac{6}{5}-\frac{3b^{2}}{20B\beta +40B\varsigma }$.

From Proposition 1, we find that the optimal misreport level $\theta_{ES}^{E*}$ is related to the unit production cost of crops *ς*, the unit cost of poverty alleviation *B*, the consumer’s price sensitivity to effort *b*. We obtain the following corollary:

**Corollary 1**: When $-3b^{2}+4\theta_{ES}^{E}B\left(\beta +2\varsigma \right)<0$, then $\frac{\partial \theta_{ES}^{E^{*}}}{\partial b}<0$, $\frac{\partial \theta_{ES}^{E^{*}}}{\partial B}>0$,$\frac{\partial \theta_{ES}^{E^{*}}}{\partial \varsigma }>0$.

According to Proposition 1 and Corollary 1, the optimal misreport level $\theta_{ES}^{E*}$ increases with an increase in investment cost *B* and production cost of farmer *ς*, but decreases with an increase in consumer price sensitivity to poverty alleviation efforts. Suppose the subsidy policy is related to the economic costs associated with the poverty alleviation of enterprises. In that case, enterprises can increase their profits within a certain range by over-reporting poverty alleviation cost information. The motivation for this behavior is that the government always provides financial support for poverty alleviation programs even under over-reporting. Therefore, enterprises can defraud the government of large amounts of subsidies by falsifying financial data and misrepresenting cost information. This behavior can also reduce the purchase price of crops, thereby increasing enterprises’ profits and reducing the impact of poverty alleviation efforts [36–38]. In practice, governments can design poverty alleviation policies by increasing public sensitivity to poverty alleviation actions or reducing enterprises and farmers’ input costs in poverty alleviation programs [39–41]. These actions can reduce the moral hazard of forging financial data and fraudulent poverty alleviation subsidy withdrawal [42–45].

### FS model

In addition to working with social enterprises, the cooperative can help farmers in many aspects, including reducing production cost, increasing process yield, boosting brand awareness, and eliminating unnecessary intermediaries [46, 47]. It is effective to study the optimum scale of the cooperative and provide a channel option scheme by comparing the cooperative channel and direct channel. If farmers participate in enterprise poverty alleviation projects, and there is motivation to report production cost information, government subsidies will not be considered.


$$Max\left(\prod_{NS}^{F}\right)=wq-\theta_{NS}^{F}\varsigma q^{2}$$ (19)

$$Max\left(\pi_{NS}^{E}\right)=\left(A+bx-\beta q-w\right)q-Bx^{2}/2$$ (20)

In the case of receiving subsidies from the government, the optimal decision-making of farmers’ open profits, enterprises, and the government are as follows:

$$Max\left(\prod_{FS}^{F}\right)=wq-\theta_{FS}^{F}\left(1-s_{FS}^{F}\right)\varsigma q^{2}$$ (21)

$$Max\left(\pi_{FS}^{E}\right)=\left(A\text{+}bx-\beta q-w\right)q-Bx^{2}/2$$ (22)

$$Max\left(\prod_{FS}^{G}\right)=\frac{\beta q^{2}}{2}+wq-\theta_{FS}^{F}\varsigma q^{2}+\left(A+bx-\beta q-w\right)q-Bx^{2}/2$$ (23)

In the above two cases, the actual profits of farmers are as follows:

$$\pi_{NS}^{F}=wq-\varsigma q^{2}$$ (24)

$$\pi_{FS}^{F}=wq-\varsigma q^{2}+\theta_{FS}^{F}s_{FS}^{F}\varsigma q^{2}$$ (25)

The inverse order is used to solve the problem when. $\theta_{FS}^{F}=1$, the profit of farmers is $\pi_{FSO}^{F}$. When $\theta_{FS}^{F}>1$, the production cost of farmers is $\pi_{FSL}^{F}$. Only if the profit $\pi_{FSL}^{F}$ with the misreported poverty alleviation cost is greater than the profit $\pi_{FSO}^{F}$ with the non-misreported poverty alleviation cost, the farmers will tend to misreport the cost. Proposition 2 and Corollary 2 are obtained as follows.

**Proposition 2**: When $-b^{2}+B\left(\beta +2\theta_{FS}^{F}\varsigma \right)>0$:

1. If the coefficient is misreported $\theta_{FS}^{F}\in \left(1,{\bar{\theta }}_{FS}^{F}\right)$, then $\pi_{FSO}^{F}<\pi_{FSL}^{F}$. If the coefficient is misreported $\theta_{FS}^{F}\geq {\bar{\theta }}_{FS}^{F}$, then $\pi_{FSO}^{F}\geq \pi_{FSL}^{F}$.
2. Misreport coefficient $\theta_{FS}^{F}\in \left[1,\theta_{FS}^{F^{*}}\right]\frac{\partial \pi_{FSL}^{F}}{\partial \theta_{FS}^{F}}>0$. If the coefficient is falsely reported $\theta_{FS}^{F}>\theta_{FS}^{F^{*}}$, then $\frac{\partial \pi_{FSL}^{F}}{\partial \theta }<0$ where the critical misreport coefficient is ${\bar{\theta }}_{FS}^{F}=\frac{3b^{4}-8b^{2}B\left(\beta +\varsigma \right)+B^{2}{\left(5\beta^{2}+10\beta \varsigma +8\varsigma \right)}^{2}}{2B^{2}\left(2\varsigma -\beta \right)\varsigma }$, and the optimal misreport coefficient is $\theta_{FS}^{F^{*}}=\frac{-3b^{2}+5B\beta +8B\varsigma }{6B\varsigma }$.

**Corollary 2**: When $-b^{2}+B\left(\beta +2\theta_{FS}^{F}\varsigma \right)>0$, $\frac{\partial \theta_{FS}^{F^{*}}}{\partial b}<0$, $\frac{\partial \theta_{FS}^{F^{*}}}{\partial B}>0$. When $b^{2}<\frac{5}{3}B\beta$, $\frac{\partial \theta_{FS}^{F^{*}}}{\partial \varsigma }>0$. When $b^{2}>\frac{5}{3}B\beta$, $\frac{\partial \theta_{FS}^{F^{*}}}{\partial \varsigma }<0$.

From Proposition 2 and Corollary 2, we find that when a farmer reports higher cost information, more profits can be obtained, and profits increase with the misreport level $\theta_{FS}^{F}$. In addition, $\partial \theta_{FS}^{F*}/\partial b<0$,$\partial \theta_{FS}^{F*}/\partial B>0$ show that the optimal misreport level $\theta_{FS}^{F*}$ increases with the increasing cost of effort *B* and decreases with increasing consumer price sensitivity to effort *b*. Obviously, increasing consumers’ acceptance of poverty alleviation products and reducing the cost of poverty alleviation programs are essential approaches to reducing farmers’ misreport levels.

### SP model

The government stands to gain from subsidy policy, such as by intervening in agricultural economic development, making up for market imperfections, and enlarging the economic scale, thereby obtaining more economic benefits. To improve agricultural production levels, subsidies are a common policy used by many countries [48–51]. Schmitz et al. show that agricultural subsidy policies in developed countries can stimulate the growth of farmers’ rents. However, the overall level of income of all farmworkers has not increased significantly [52]. Although higher cost information misreported by enterprises and farmers can improve their interests, it would result in loss to other supply chain members.

To mitigate this undesired outcome, the local government should reduce the level of asymmetry in cost information and further improve poverty alleviation efficiency by taking measures, such as supervision and punitive mechanisms associated with misreporting behaviors. To investigate this problem, we make the following assumptions: the level of government supervision over the enterprise is *k*(0 < *k* ≤ 1), and its cost coefficient and penalty coefficient are *k* and *γ*, respectively. The following is an example of an enterprise’s misreporting model.

Decision-making by farmers is stated as:

$$Max\left(\pi_{SP}^{F}\right)=wq-\varsigma q^{2}$$ (26)

The public profit of the enterprise is stated as:

$$Max\left(\prod_{SP}^{E}\right)=\left\{\begin{array}{ll} k\left(1-\gamma \right)\left(\Lambda -\frac{\theta_{SP}^{E}Bx^{2}}{2}\right)+\left(1-k\right)\left[\Lambda -\frac{\theta_{SP}^{E}Bx^{2}}{2}\left(1-s_{SP}^{E}\right)\right] & ,\;\theta_{SP}^{E}>1\\ \Lambda -\frac{Bx^{2}}{2}\left(1-s_{SP}^{E}\right) & ,\;\theta_{SP}^{E}=1 \end{array}\right.$$ (27)

Government decision-making is represented as:

$$Max\left(\pi_{SP}^{G}\right)=\left\{\begin{array}{ll} \frac{\beta q^{2}}{2}+\pi_{SP}^{F}+\prod_{SP}^{E}+k\gamma \left(\Lambda -\frac{\theta_{SP}^{E}Bx^{2}}{2}\right)-\left(1-k\right)s_{SP}^{E}\frac{\theta_{SP}^{E}Bx^{2}}{2}-kx & ,\;\theta_{SP}^{E}>1\\ \frac{\beta q^{2}}{2}+\prod_{SP}^{E}+\pi_{SP}^{F}-s_{SP}^{E}\frac{Bx^{2}}{2}-kx & ,\;\theta_{SP}^{E}>1 \end{array}\right.$$ (28)

The actual profit of the enterprise is:

$$Max\left(\pi_{SP}^{E}\right)=\left\{\begin{array}{ll} k\left(1-\gamma \right)\left(\Lambda -\frac{Bx^{2}}{2}\right)+\left(1-k\right)\left[\Lambda -\frac{Bx^{2}}{2}+\theta_{SP}^{E}\frac{Bx^{2}}{2}s_{SP}^{E}\right] & ,\theta_{SP}^{E}>1\\ \Lambda -\frac{Bx^{2}}{2}\left(1-s_{SP}^{E}\right) & ,\;\theta_{SP}^{E}=1 \end{array}\right.$$ (29)

where, Λ = (*A* + *bx* − *βq* − *w*)*q*.

## Results and discussion

In the previous sections, we studied four poverty-alleviation supply chain models. This section compares the results from two perspectives: the coefficients of government subsidies and punitive measures policy. First, we discuss the relationship between farmers’ profit, enterprise profit, total government subsidy input, social welfare, and misrepresentation level.

**Proposition 3**: Under the act of misrepresentation, the following holds:

1. $\frac{\partial \pi_{ES}^{F^{*}}}{\partial \theta_{ES}^{E}}<0$, $\frac{\partial \pi_{ES}^{G^{*}}}{\partial \theta_{ES}^{E}}<0$, $\frac{\partial \pi_{FS}^{E^{*}}}{\partial \theta_{FS}^{F}}<0$, $\frac{\partial \pi_{FS}^{G^{*}}}{\partial \theta_{FS}^{F}}<0$.
2. $\frac{\partial TS_{ES}^{*}}{\partial \theta_{ES}^{E}}<0$, $\frac{\partial TS_{FS}^{*}}{\partial \theta_{FS}^{F}}<0$.
3. $\Delta \pi_{ES}^{F}>0$, $\Delta \pi_{ES}^{G}>0$, $\frac{\partial \Delta \pi_{ES}^{F}}{\partial \theta_{ES}^{E}}<0$, $\frac{\partial \Delta \pi_{ES}^{G}}{\partial \theta_{ES}^{E}}<0$, $\Delta \pi_{FS}^{E}>0$,$\Delta \pi_{FS}^{G}>0$,$\frac{\partial \Delta \pi_{ES}^{E}}{\partial \theta_{ES}^{F}}<0$,$\frac{\partial \Delta \pi_{FS}^{G}}{\partial \theta_{ES}^{F}}<0$.

Hence, $\Delta \pi_{ES}^{F}=\pi_{ES}^{F^{*}}-\pi_{NS}^{F^{*}}$,$\Delta \pi_{ES}^{G}=\pi_{ES}^{G^{*}}-\pi_{NS}^{G^{*}}$,$\Delta \pi_{ES}^{E}=\pi_{ES}^{G^{*}}-\pi_{NS}^{G^{*}}$,$\Delta \pi_{FS}^{G}=\pi_{FS}^{G^{*}}-\pi_{NS}^{G^{*}}$.

From Proposition 3, we find that government subsidies can effectively improve enterprises and farmers’ profits and total social welfare. It can also help farmers get rid of poverty and create more value. However, suppose the above two sides conceal the true financial cost information in the process of poverty alleviation. In that case, they will reduce their enthusiasm to participate in poverty alleviation projects and negatively impact other participants. For example, when an enterprise overstates the poverty alleviation cost, open profit will decrease, and the enterprise will reduce agricultural products’ purchase price. As a result, farmers’ enthusiasm for products will be dampened, and even worse, their profit would fall: $\frac{\partial \pi_{ES}^{F^{*}}}{\partial \theta_{ES}^{E}}<0$. For the government, we obtain the results that $\frac{\partial \Delta \pi_{ES}^{G}}{\partial \theta_{ES}^{E}}<0$,$\frac{\partial TS_{ES}^{*}}{\partial \theta_{ES}^{E}}<0$. This shows that the increase in social welfare brought by financial subsidies will decline with an increase in the misreporting level. Under the condition that enterprises generally misrepresent the cost information of poverty alleviation, their enthusiasm to participate in poverty alleviation activities will decrease, and the government’s total subsidy input will also decrease accordingly.

We now compare the efficiency of poverty alleviation under the two subsidy modes of the NS and ES models. Following Berenguer et al. [36], we assume that the efficiency of poverty alleviation subsidy per unit is higher than that of subsidy $\eta =\frac{\Delta \pi^{G}}{TS}$, which is the ratio of social welfare growth to the total government subsidy input.

**Proposition 4**: Under the situation of misrepresentation, $\frac{\partial \eta_{ES}}{\partial \theta_{ES}^{E}}<0$, $\frac{\partial \eta_{FS}}{\partial \theta_{FS}^{F}}>0$.

It is worth noting that misreporting of cost information by enterprises or farmers has a negative impact on the increment of social welfare and the total input of subsidies. Moreover, the variation trend of subsidy efficiency per unit is not consistent with that of misreporting. When the government provides subsidies for enterprises’ poverty alleviation, with an increase in enterprises’ misreporting, the efficiency of subsidy shows a decreasing trend. If the government provides subsidies for farmers’ participation and cooperation, with an increase in farmers’ misreporting, the efficiency of subsidy will show an increasing trend. As the government directly grants subsidies to farmers, an increase in the level of farmers’ misreporting leads to an increase in the purchase price of agricultural products, the enthusiasm for production decreases, and the consumer surplus correspondingly decreases. Precious studies have argued that when the income from selling agricultural products decreases significantly, and the total amount of decrease is higher than the total increase in farmers’ profits, social welfare is reduced [24, 53, 54]. Because subsidy is a quadratic function with respect to output *q*, the decline in enthusiasm for production has a great negative impact on the total amount of subsidies. Thus, the total subsidy funds obtained are rapidly reduced. Considering that $\frac{\partial \Delta \pi_{FS}^{G}}{\partial \theta_{FS}^{F}}<\frac{\partial TF_{FS}}{\partial \theta_{FS}^{F}}$, we can conclude that the decremental decrease in social welfare is slower than that of the total subsidy input. The efficiency of subsidy is positively correlated with the level of misreporting. In practice, it is common for farmers to report production cost information and falsely claim subsidies. Due to the decrease in total subsidy amount for local governments, even as the efficiency of subsidy increases, some countries have not implemented strict control and management of this phenomenon [55, 56].

Next, we compare the efficiency of subsidies by using the two subsidy modes in misrepresentation and obtain Proposition 5.

**Proposition 5**: Under the act of misrepresentation,

1. When $\varsigma >\frac{3b^{2}}{4B}-\frac{\beta }{2}$, *η*_*FS*_ > *η*_*ES*_.
2. When $\varsigma >\frac{3b^{2}}{4B}-\frac{\beta }{2}$. If $\theta_{ES}^{E}$ and $\theta_{FS}^{F}$ meets the following:
$$\left\{\begin{array}{c} \frac{2\theta_{ES}^{E}T_{1}\left[-3b^{2}+4\theta_{ES}^{E}B\left(\beta +2\varsigma \right)\right]}{{\left[b^{2}-2\theta_{ES}^{E}B\left(\beta +2\varsigma \right)\right]}^{2}}+\frac{3b^{4}}{{\left[b^{2}-2B\left(\beta +2\theta_{FS}^{F}\varsigma \right)\right]}^{2}}>3\\ \theta_{ES}^{E}\geq 1,\;\theta_{FS}^{F}\geq 1 \end{array}\right.,$$
  then *η*_*ES*_ > *η*_*FS*_.

From Proposition 5, the government should choose different subsidy methods in different situations. If the farmers’ planting cost coefficient is large (*ς* > 3*b*^2^/4*B* − *β*/2), it is necessary to invest more in planting crops per area *η*_*FS*_ > *η*_*ES*_. Government subsidies directly based on farmers’ planting situation can reduce farmers’ economic pressure and obtain higher subsidy efficiency. If the farmer planting cost coefficient is small (*ς* < 3*b*^2^/4*B* − *β*/2), we will also consider the situation:$\theta_{ES}^{E}=\theta_{FS}^{F}=1$ where both the farmers and the enterprises disclose the cost information. Due to the economic pressure faced by farmers in production activities, the government provides financial subsidies according to the cost expenditure of poverty alleviation of enterprises, which can more effectively promote poverty alleviation and improve the efficiency of subsidy policies [57, 58].

Only if the enterprise’s expected profit misreporting the cost $\left(\pi_{SP}^{E}|\theta_{SP}^{E}>1\right)$ is less than the expected profit of disclosing the cost $\left(\pi_{SP}^{E}|\theta_{SP}^{E}=1\right)$ can the government’s punitive measures be effective. Consequently, we propose Proposition 6.

**Proposition 6**: When $\gamma >\frac{3\left(\theta_{SP}^{E}-1\right)b^{2}\left[3b^{2}+4\left(2\theta_{SP}^{E}-3\right)B\left(\beta \text{+}2\varsigma \right)\right]}{k\left[3b^{2}-4B\left(\beta \text{+}2\varsigma \right)\right]\left[3\left(-3+\theta_{SP}^{E}\right)b^{2}+8{\left(\theta_{SP}^{E}\right)}^{2}B\left(\beta \text{+}2\varsigma \right)\right]}$, it can be obtained that $\left(\pi_{SP}^{F^{*}}|\theta_{SP}^{E}>1\right)<\left(\pi_{SP}^{F^{*}}|\theta_{SP}^{E}=1\right)$.

If punitive measures are effective, enterprises will take the initiative to disclose the cost of poverty alleviation $\theta_{SP}^{E}=1$. Comparing the profit and subsidy input of enterprises, farmers, and the government before and after the implementation of the punitive measures, we obtain:

**Corollary 3**: When the government’s punitive measures are effective, $\pi_{SP}^{E^{*}}>\pi_{ES}^{E^{*}}$, $\pi_{SP}^{F^{*}}>\pi_{ES}^{F^{*}}$, $TS_{SP}^{*}<TS_{ES}^{*}$, only if the government supervises and the cost input *k* is satisfied that $k<\frac{9\left(\theta_{SP}^{E}-1\right)A^{2}b^{2}B}{2\left[3b^{2}-4B\left(\beta +2\varsigma \right)\right]\left[3b^{2}-4\theta_{SP}^{E}B\left(\beta +2\varsigma \right)\right]}$, $\pi_{SP}^{G^{*}}>\pi_{ES}^{G^{*}}$ will be established.

Proposition 6 and Corollary 3 show that implementing the punitive measures policy within a certain range for misreporting can promote enterprises to reduce the level of misreporting of cost information. When the government’s punitive measures for misrepresentation increase, the expected profit of enterprises in overstating the cost of poverty alleviation information is less than that of disclosing the cost of information; therefore, enterprises will choose to disclose the actual cost of poverty alleviation [59]. Wang and Li found that the total amount of subsidy for poverty alleviation in implementing both supervisory and punitive measures policies is lower than that of implementing only the subsidy policy [18]. However, some scholars argue that the farmers’ profit and the enterprise’s profit will be higher in implementing the subsidy policy [16, 40].

Moreover, when the supervision cost is controlled within a certain range, social welfare will increase [45]. Therefore, for the government, the formulation of appropriate supervision and punitive measures can regulate enterprises’ behavior and reduce the degree of information asymmetry in the poverty alleviation supply chain. Furthermore, it can effectively safeguard the interests of the poor and improve the overall efficiency of the poverty alleviation supply chain.

## Numerical analysis

Numerical analyses were carried out on the results of the above model. Following Pu et al. [60], we assume the potential size of the market *A* = 100, poverty alleviation cost coefficient *B* = 1, consumers of poverty alleviation action sensitive coefficient *b* = 1, consumer price-sensitive coefficient *β* = 1/2, and cooperative production plant cost coefficient *ς* = 2. This section discusses the influence of the change in misstatement level and government subsidy coefficient on farmers, enterprises, and governments’ decision-making and income under different circumstances.

### Effects of misreporting behavior on the profit of supply chain members

When an enterprise conceals the true information of poverty alleviation costs, the change in profit associated with enterprises, farmers, government, and the total subsidy under the misreport level *θ* are shown in Fig 1a. When the misreport level changes *θ* from 1 to 2, the profit of the enterprise increases first and then decreases. The enterprise profit reaches the highest level of 671.3 at the optimal misreport level $\theta_{ES}^{E}=1.17$. However, if the misreport level continues to increase, enterprise profit will decrease. Specifically, when the misreport level $\theta_{ES}^{E}>1.45$, the enterprise profit is below 664.5, which is even less than the normal reporting situation. This tendency indicates that this enterprise could not always benefit from misreporting higher cost information, although this misreporting behavior brings in more subsidies from the government. Simultaneously, the profits of farmers, total social welfare, and total subsidies decrease with increasing misreporting levels *θ*. This implies that enterprises’ misreporting behavior would always damage both the farmers’ and the government’s profits. Worse, the poverty problems in rural areas remain unsolved. To avoid this undesired consequence, the government should screen enterprises’ misreporting behavior by implementing supervisory and punitive policies when promoting more poverty alleviation programs in poor areas [61]. For example, the Chinese State Council Poverty Alleviation and Development Group pointed out that, in 2017, the abuse of poverty alleviation funds or marketing activities in the name of poverty alleviation would be resolutely investigated, the miscreants punished, and severe punitive measures imposed on improper behaviors in the process of poverty alleviation to standardize poverty alleviation projects.

**Fig 2:**
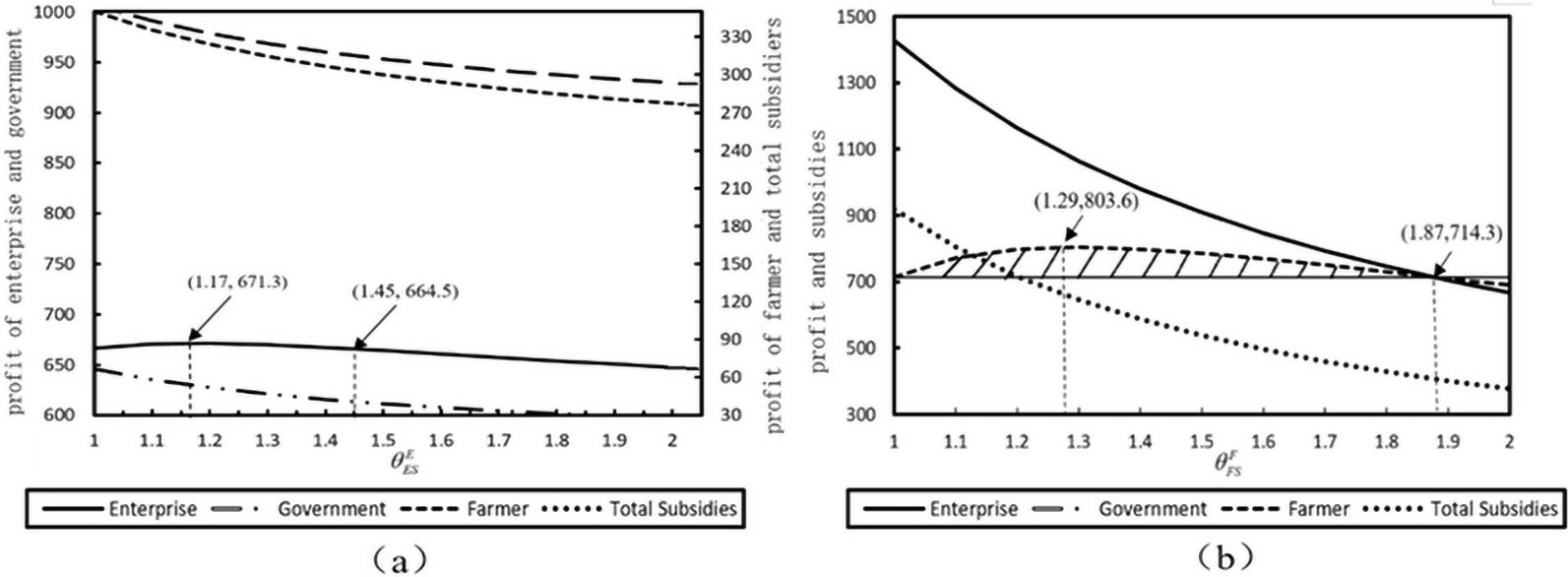
The impact of misreporting level on the profit of members in the supply chain.

Similar to Fig 2a and 2b shows the situation where the farmer misreports its cost information. This misreporting behavior would also hurt enterprises and governments’ benefits, thus decreasing the government’s total subsidies. Consequently, poverty alleviation projects fail to achieve the expected results.

### Comparison of subsidy efficiency in two situations

**Fig 3:**
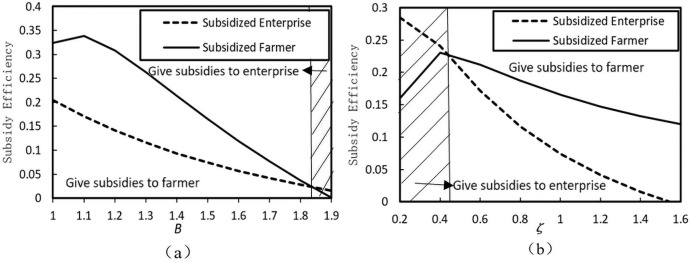
The impact of cost coefficient on the subsidy efficiency.

By assuming *β* = 1, *ς* = 1, *b* = 1.5, we draw Fig 3a, discussing the impact of the poverty alleviation cost coefficient *B* on the subsidy efficiency under the two subsidy policies. Fig 3a shows that when *B* = 1, the result of *η*_*ES*_ = 0.21 < *η*_*FS*_ = 0.32 implies that the efficiency of subsidizing farmers is higher than that of subsidizing enterprises. As *B* increases, *η*_*ES*_ decreases but *η*_*FS*_ increases first and then decreases. When *B* = 1.84, the efficiency of subsidizing farmers is equal to that of subsidizing enterprises, that is, *η*_*ES*_ = *η*_*FS*_ = 0.30. When *B* continues to increase, this efficiency is higher than when subsidizing farmers. In addition, the figure shows that the total amount of subsidies issued by the government is less than an increment in social welfare. This is due to the “deadweight loss effect,” according to Arya and Mittendorf [7]. In other words, the government’s financial subsidy would not translate into social welfare. We conclude that enterprises’ higher cost of poverty alleviation programs will result in large economic pressure. Xing et al. believed that the government should directly provide subsidy funds to enterprises [40]. Under this subsidy, to relieve this pressure, enterprises will have more enthusiasm to participate in poverty alleviation projects and help farmers in poor areas.

By assuming *β* = 1, *b* = 1, *B* = 1.5, we draw Fig 3b, where the impact of the production cost coefficient of the farmer *ς* on the subsidy efficiency of the two subsidy policies is analyzed. If the government provides subsidies to farmers, the efficiency of subsidy policy increases first and decreases with an increase in *ς*. In contrast, the efficiency of subsidized enterprises monotonically decreases with an increase in *ς*. When *ς* < 0.47, subsidized enterprises are more effective than subsidized farmers. However, when *ς* > 0.47, an opposite result occurs. From what we have discussed, it is better to give subsidies to farmers directly under a high production cost while providing subsidies to enterprises under a low production cost.

Therefore, in poverty alleviation programs, the government should choose an appropriate subsidy model depending on the input cost level of both sides to obtain higher subsidy efficiency.

### Effects of supervision and punitive measures on optimal decision

**Fig 4:**
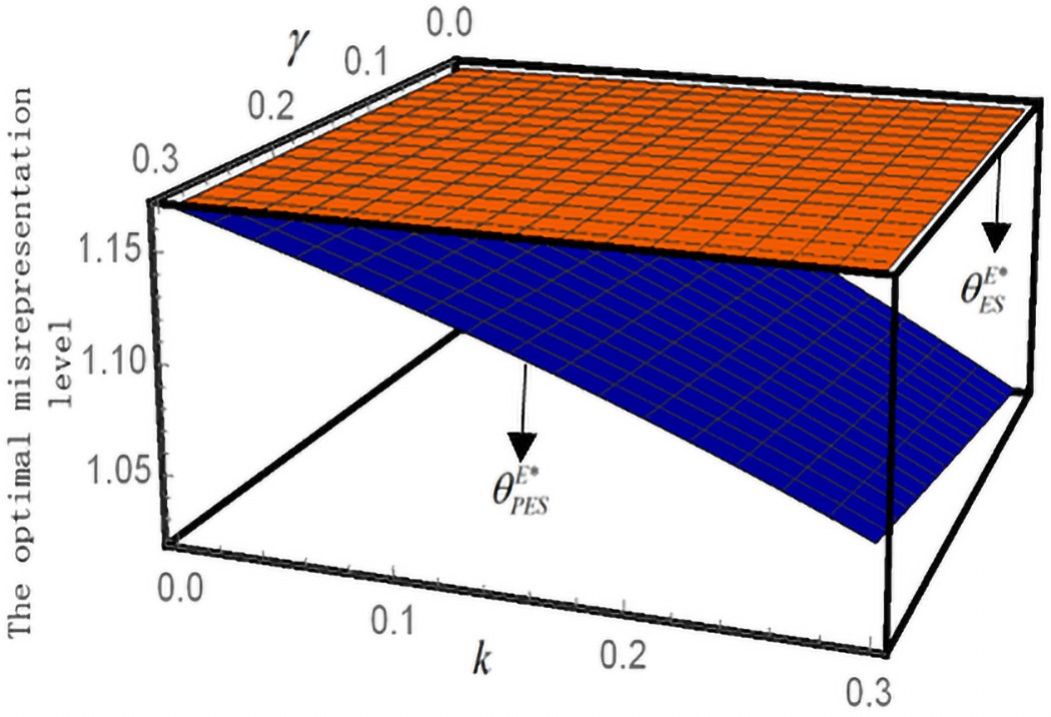
The impact of *k* and *γ* on the optimal misreport level.

By assuming *b* = 1, *B* = 1, *ς* = 1.6, and *β* = 1.5, Fig 4 shows the impact of *γ* and *k* on the optimal misreport level $\theta_{SP}^{E^{*}}$. $\theta_{ES}^{E^{*}}<\theta_{SP}^{E^{*}}=1.168$ implies that the optimal misreport level $\theta_{SP}^{E^{*}}$ under the punitive measures policy is lower than $\theta_{ES}^{E^{*}}$. The result indicates that punitive measures can effectively alleviate an enterprise’s misreporting behaviors. Meanwhile, $\theta_{SP}^{E^{*}}$ increases with an increase in the penalty coefficient *γ* and decreases with an increase in the supervision level *k*. This is consistent with the conclusion in Proposition 5. This result is consistent with the conclusions of Yi et al. [62]. When the government fails to carry out effective subsidy supervision, enterprises will increase their misreporting costs to fraudulently claim subsidies. From an institutional perspective, strict approval procedures should be implemented for the acquisition of poverty alleviation subsidies [63]. However, Meng found that many local township governments, which assume audit responsibility, tend to be formal and not careful enough in inspection [64]. As long as they see the appraisal opinions stamped with the official seal, the first test is passed. They will no longer seriously check the subsidized enterprises and farmers, implying there is no effective supervision.

**Fig 5:**
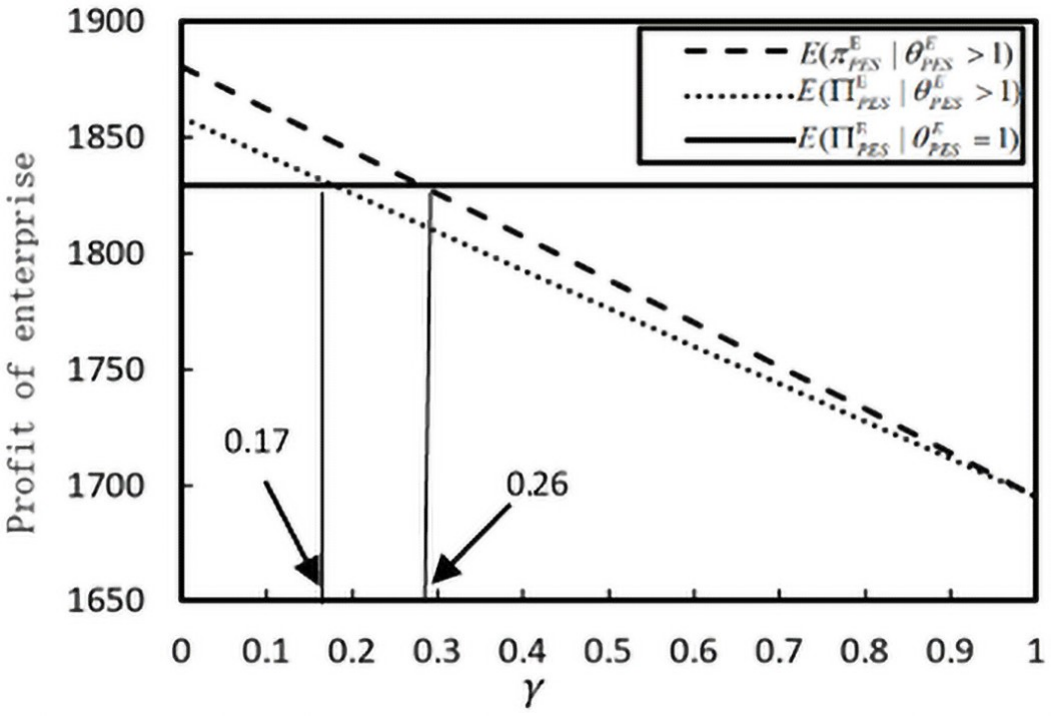
The impact of *γ* on the profit of enterprise.

We assume *b* = 2, *B* = 0.9, *ς* = 1.6, *β* = 1.5, *k* = 0.1. Fig 5 shows the impact of *k* on enterprise profit. Both the expected public profit of enterprise $E\left(\prod_{SP}^{E}|\theta_{SP}^{E}>1\right)$ and the corresponding real profit decrease $E\left(\pi_{SP}^{E}|\theta_{SP}^{E}>1\right)$ with increasing *γ*. Note that, under-reporting the real cost information, the enterprise’s expected profit $E\left(\prod_{SP}^{E}|\theta_{SP}^{E}=1\right)$ is equal to 1829.27. Further, we have $E\left(\pi_{SP}^{E}|\theta_{SP}^{E}>1\right)<E\left(\prod_{SP}^{E}|\theta_{SP}^{E}=1\right)$, when *γ* > 0.26.$E\left(\prod_{SP}^{E}|\theta_{SP}^{E}>1\right)<E\left(\prod_{SP}^{E}|\theta_{SP}^{E}=1\right)$, when *γ* > 0.17. According to Proposition 6, the punitive measures policy is invalid if *γ* < 0.17. As the penalty coefficient *γ* increases, the drawbacks of misreporting behavior become apparent, and the enterprise profit decreases. Thus, to standardize enterprises’ performance in implementing poverty alleviation programs, the government should supervise and check the enterprise information and then impose penalties on enterprises that violate the regulations.

**Fig 6:**
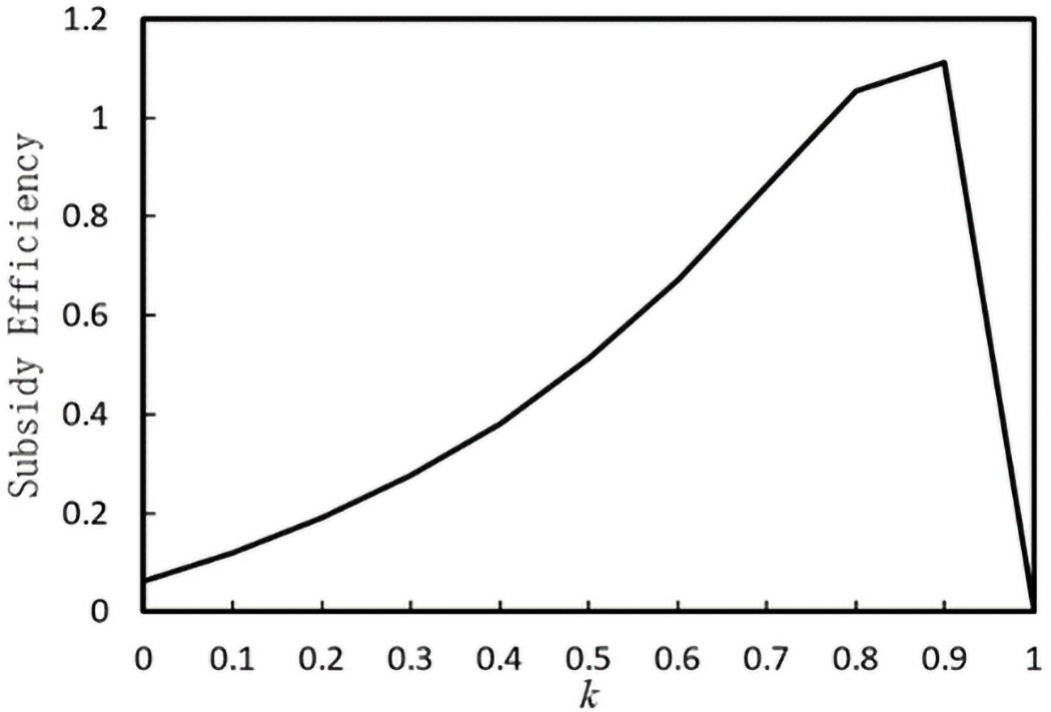
The impact of *k* on the subsidy efficiency.

Under the assumption of *b* = 1.5, *B* = 1, *ς* = 1.6, *β* = 1.5, *γ* = 0.3, *k* = 10, Fig 6 shows the impact of *k* on the subsidy efficiency. This efficiency decreases first and then increases with increasing *k*. This is because, under a small government supervision level *k*, both the probability of an enterprise’s misreporting behavior and the total fines increase with an increase *in k*. A change in total social welfare is greater than that in total subsidies. Therefore, subsidy efficiency shows an increasing trend. A high supervision level chosen by the government means a higher supervision cost. This will reduce total social welfare, which is greater than the reduction in total subsidies. Thus, this efficiency is a decreasing trend. Krafft stated that these behaviors seriously harm the interests of poor farmers and are not conducive to the sustainable promotion of poverty alleviation policies [65]. In the practice of poverty alleviation, the government should mobilize social forces to publicize the efforts, reduce the cost of establishing a poverty alleviation supply chain between enterprises and farmers, and increase consumers’ attention to poverty alleviation projects [66]. Simultaneously, corresponding monitoring–punishment measures should be formulated for farmers and enterprises to cheat financial subsidies by misreporting cost information. Sampling inspection should be conducted on the financial situation of enterprises’ poverty alleviation projects and farmers’ participation in poverty alleviation projects to restrain the subsidized subjects’ misreporting behavior [67].

## Conclusion

We analyze the misreporting behavior of supply chain members under subsidy policy in poverty alleviation programs. These misreporting behaviors are significant for both enterprises and farmers. The first situation considers that the government subsidizes enterprises according to its poverty alleviation cost, and the enterprise has the motivation to report a higher cost coefficient to obtain more subsidies. The second situation considers that the government provides subsidies to the farmer and his misreporting behavior over cost information. Based on the two situations above, we propose a supervision and punishment policy and examine its effectiveness.

The model yields the following insights: (1) Enterprises and farmers can increase their profits by reporting higher cost information within a certain range. However, this misreporting behavior always damages other supply chain participants’ interests and is not conducive to the promotion of poverty alleviation projects. (2) The optimal misreporting level increases with both the unit cost of poverty alleviation efforts and the unit production cost of farmers but decreases with the consumer’s price sensitivity to poverty alleviation efforts. (3) When the cost factor of poverty alleviation efforts from enterprises is small, the government should provide subsidies to farmers; when production cost factors from farmers are small, the government should provide subsidies to enterprises. (4) Although the government can use supervision and punishment policy to constrain misreporting behavior effectively, the additional costs incurred in the progress of supervision behavior would cause an uncertain effect on the efficiency of the subsidy policy.

However, the model in this study has some limitations. First, we do not consider how the crop output would change in different climates and other factors. Second, the study assumes that a social enterprise’s poverty alleviation effort would have a linear impact on-demand function rather than other forms. Therefore, future research will concentrate on these scenarios so that relevant results more closely match reality.

## Supporting information

S1 Data (DOCX)
