# Supplementary material for: Strategies for poverty alleviation supply chain with government subsidies and misreporting behavior in China
Source: PLoS One. 2021 Jul 15;16(7):e0253761. doi: 10.1371/journal.pone.0253761 (PMC8282072; doi:10.1371/journal.pone.0253761)
Supplement: S1 Data — (DOCX) [file pone.0253761.s001.docx]

| **Fig 2: The impact of misreporting level on the profit of members in the supply chain** | | | | |
| --- | --- | --- | --- | --- |
| $\theta_{ES}^{E}$ | Enterprise | Government | Farmer | Total Subsidies |
| 1.0 | 668.7 | 648.7 | 1100.4 | 1000.2 |
| 1.1 | 670.2 | 642.3 | 991.7 | 985.5 |
| 1.2 | 672.3 | 638.1 | 984.1 | 978.6 |
| 1.3 | 669.5 | 625.8 | 977.5 | 968.4 |
| 1.4 | 665.8 | 623.4 | 968.4 | 952.8 |
| 1.5 | 663.7 | 618.2 | 965.3 | 942.2 |
| 1.6 | 662.2 | 610.4 | 960.6 | 937.9 |
| 1.7 | 660.1 | 605.1 | 958.2 | 925.3 |
| 1.8 | 658.3 | 601.3 | 955.5 | 918.6 |
| 1.9 | 655.6 | 600.5 | 953.4 | 910.8 |
| 2.0 | 652.2 | 600.2 | 948.7 | 905.6 |
| $\theta_{FS}^{E}$ | Enterprise | Government | Farmer | Total Subsidies |
| 1.0 | 1460.1 | 701.2 | 701.2 | 901.8 |
| 1.1 | 1302.6 | 705.6 | 730.8 | 749.6 |
| 1.2 | 1160.5 | 704.3 | 742.1 | 702.8 |
| 1.3 | 1009.5 | 712.2 | 750.6 | 680.4 |
| 1.4 | 960.7 | 713.4 | 741.3 | 657.1 |
| 1.5 | 912.6 | 716.4 | 736.8 | 625.3 |
| 1.6 | 903.7 | 715.2 | 729.4 | 579.2 |
| 1.7 | 870.2 | 715.1 | 720.8 | 518.6 |
| 1.8 | 710.1 | 715.2 | 712.5 | 496.5 |
| 1.9 | 703.3 | 714.5 | 702.1 | 453.7 |
| 2.0 | 680.4 | 716.8 | 700.6 | 401.6 |

| **Fig 3: The impact of cost coefficient on the subsidy efficiency** | | |
| --- | --- | --- |
| B | Subsidized Enterprise | Subsidized Farmer |
| 1 | 0.20469 | 0.32432 |
| 1.1 | 0.17136 | 0.33819 |
| 1.2 | 0.14192 | 0.30858 |
| 1.3 | 0.11621 | 0.26339 |
| 1.4 | 0.09375 | 0.21403 |
| 1.5 | 0.07407 | 0.16532 |
| 1.6 | 0.05674 | 0.11924 |
| 1.7 | 0.04140 | 0.07648 |
| 1.8 | 0.02773 | 0.03716 |
| 1.9 | 0.01548 | 0.00115 |
| $\varsigma$ | Subsidized Enterprise | Subsidized Farmer |
| 0.2 | 0.28478 | 0.16011 |
| 0.4 | 0.24091 | 0.23082 |
| 0.6 | 0.17136 | 0.21231 |
| 0.8 | 0.11621 | 0.18726 |
| 1 | 0.07407 | 0.16532 |
| 1.2 | 0.04140 | 0.14724 |
| 1.4 | 0.01548 | 0.13240 |
| 1.6 | -0.00551 | 0.12012 |

| **Fig 4.** The impact of *k* and $\gamma$on the optimal misreport level | | | |
| --- | --- | --- | --- |
| *k* | misreport level | $\gamma$ | misreport level |
| 0 | 1.16809 | 0 | 1.12517 |
| 0.1 | 1.16809 | 0.1 | 1.12930 |
| 0.2 | 1.16809 | 0.2 | 1.13346 |
| 0.3 | 1.16809 | 0.3 | 1.13766 |
| 0.4 | 1.16809 | 0.4 | 1.14189 |
| 0.5 | 1.16809 | 0.5 | 1.14616 |
| 0.6 | 1.16809 | 0.6 | 1.15047 |
| 0.7 | 1.16809 | 0.7 | 1.15482 |
| 0.8 | 1.16809 | 0.8 | 1.15920 |
| 0.9 | 1.16809 | 0.9 | 1.16362 |
| 1 | 1.16809 | 1 | 1.16809 |

| **Fig 5.** The impact of $\gamma$ on the profit of enterprise | | | |
| --- | --- | --- | --- |
| $E\left(\prod_{SP}^{E}\vert\theta_{SP}^{E}>1\right)$ | $E\left(\pi_{SP}^{E}\vert\theta_{SP}^{E}>1\right)$ | $E\left(\prod_{SP}^{E}\vert\theta_{SP}^{E}=1\right)$ | $\gamma$ |
| 1880.66 | 1858.70 | 1829.27 | 0 |
| 1862.35553 | 1842.11313 | 1829.27 | 0.1 |
| 1843.98860 | 1825.57430 | 1829.27 | 0.2 |
| 1825.56081 | 1809.08353 | 1829.27 | 0.3 |
| 1807.07375 | 1792.64080 | 1829.27 | 0.4 |
| 1788.52903 | 1776.24613 | 1829.27 | 0.5 |
| 1769.92826 | 1759.89950 | 1829.27 | 0.6 |
| 1751.27303 | 1743.60093 | 1829.27 | 0.7 |
| 1732.56494 | 1727.35040 | 1829.27 | 0.8 |
| 1713.80560 | 1711.14793 | 1829.27 | 0.9 |
| 1694.99661 | 1694.99350 | 1829.27 | 1 |

| **Fig 6.** The impact of *k* on the subsidy efficiency | |
| --- | --- |
| k | subsidy efficiency |
| 0 | 0.06232 |
| 0.05 | 0.08935 |
| 0.1 | 0.11928 |
| 0.15 | 0.15245 |
| 0.2 | 0.18923 |
| 0.25 | 0.23006 |
| 0.3 | 0.27539 |
| 0.35 | 0.32576 |
| 0.4 | 0.38173 |
| 0.45 | 0.44391 |
| 0.5 | 0.51290 |
| 0.55 | 0.58920 |
| 0.6 | 0.67313 |
| 0.65 | 0.76448 |
| 0.7 | 0.86193 |
| 0.75 | 0.96182 |
| 0.8 | 1.05538 |
| 0.85 | 1.12206 |
| 0.9 | 1.11181 |
| 0.95 | 0.88892 |
| 1 | 0 |
